# Cyclic GMP dependent protein kinase (PKG) as a mediator of EGFR-induced apoptosis in breast cancer

**DOI:** 10.1186/2050-6511-16-S1-A60

**Published:** 2015-09-02

**Authors:** Nicole M Jackson, Brian P Ceresa

**Affiliations:** 1 Department of Pharmacology and Toxicology, University of Louisville, Louisville, KY 40202, USA

## Background

The Epidermal Growth Factor Receptor (EGFR) is a transmembrane receptor tyrosine kinase with critical implications in cell proliferation, migration, wound healing and the regulation of apoptosis. However, the EGFR has been shown to be hyper-expressed in a number of human malignancies. The MDA-MB-468 metastatic breast cell line is one example of this. This particular cell line hyper-expresses the EGFR and undergoes EGFR mediated apoptosis in response to EGF ligand. Previous studies within our laboratory have shown that when the EGFR is activated and retained at the cell membrane, MDA-MB-468 cells undergo cell growth. Conversely, once the activated EGFR is internalized within endosomes, the cells undergo apoptosis [1]. This contrasting response at subcellular locations is defined as spatial regulation. The goal of this project is to identify the kinases responsible for the induction of apoptosis from within the endosomes. Research has shown that cGMP dependent protein kinase (PKG) activation within this cell line induces apoptosis in a dose dependent manner [2]. We hypothesize that PKG plays an intermediate role in the EGFR induction of apoptosis.

## Conclusion

The data confirm that PKG activation induces cell death within the MDA-MB-468 cell line. PKG activity is also correlated to, and downstream of EGFR activation. Future studies entail inhibiting PKG activity in order to determine its individual role in mediating EGFR-induced apoptosis.
